# DEL‐1 Protects the Myocardium From Ischemia/Hypoxia Injury Through Regulating the Sirt1/NF‐κB Signaling Pathway

**DOI:** 10.1002/iid3.70288

**Published:** 2025-10-28

**Authors:** Bin Hu, Wenlong Zhang, Wan‐Cheng Yu, Hao Lin

**Affiliations:** ^1^ Department of Cardiovascular Surgery Shandong Provincial Hospital Affiliated to Shandong First Medical University Jinan Shandong China

**Keywords:** apoptosis, DEL‐1, inflammation, myocardial infarction, Sirt1/NF‐κB pathway

## Abstract

**Purpose:**

Our study intended to explore the function and the potential mechanisms of developmental endothelial locus‐1 (DEL‐1) to alleviate myocardial infarction (MI).

**Methods:**

First, adeno‐associated viral containing DEL‐1 cDNA was injected into the myocardium of SD rats, and pcDNA‐DEL‐1 or Sirt1 siRNA was transfected into cardiomyocytes. Next, an MI model in vivo and a hypoxia model in vitro were established. The expression of DEL‐1 in MI rats and hypoxia‐induced cardiomyocytes was detected by qRT‐PCR, Western blot analysis, and immunofluorescence staining. The functions of DEL‐1 in vivo were investigated utilizing qRT‐PCR, echocardiography, hematoxylin and eosin staining, Masson's trichrome staining, Western blot analysis, TUNEL staining, and detection of hemodynamics, lactate dehydrogenase, and creatine kinase‐MB. The functions of DEL‐1 in vitro were investigated utilizing qRT‐PCR, Western blot analysis, ELISA, flow cytometry, co‐culture of cardiac cardiomyocytes and fibroblasts, transwell assay, and immunofluorescence staining.

**Results:**

DEL‐1 expression was downregulated in MI rats and hypoxia‐induced cardiomyocytes. DEL‐1 overexpression alleviated cardiac dysfunction, myocardial fibrosis, inflammation, and cardiomyocyte apoptosis in MI rats. In vitro, DEL‐1 overexpression alleviated cardiomyocyte apoptosis and inflammation. Moreover, we also confirmed that DEL‐1 secreted by cardiomyocytes activated cardiac fibroblasts through paracrine signaling. Besides, DEL‐1 regulated the Sirt1/NF‐κB pathway in vitro and in vivo. However, the downregulation of Sirt1 reversed the effect of DEL‐1 on cardiomyocyte apoptosis and inflammation.

**Conclusion:**

DEL‐1 could alleviate myocardial damage induced by MI via regulating the Sirt1/NF‐κB signaling pathway.

**Abbreviations**
AAV: adeno‐associated viral
CK‐MB: creatine kinase‐MB
DBP: diastolic blood pressure
DEL‐1: developmental endothelial locus‐1
EF: ejection fraction
FS: fractional shortening
LDH: lactate dehydrogenase
MAP: mean arterial pressure
MI: myocardial infarction
NF‐κB: nuclear factor‐kappaB
SBP: systolic blood pressure
Sirt1: sirtuin 1

## 1 Introduction

Myocardial infarction (MI) is regarded as a leading cause of death and loss of years of productive life all over the world [1, 2]. MI can lead to ventricular remodeling involving cellular signaling modulation and myocardial fibrosis, which is mainly due to an imbalance between myocardial blood supply and oxygen demand, and eventually progresses to heart failure [3, 4, 5]. In recent years, the morbidity and mortality of MI have been high in China, and the age of onset tends to be younger [6]. At present, the treatment of MI mainly includes removing coronary artery obstruction by surgery or antithrombolytics, but these strategies cannot reverse the irreversible myocardial damage that has occurred [7]. Hence, exploring the exact mechanism of MI development and looking for the early diagnostic markers and therapeutic targets for MI are of great significance.

The pathophysiological mechanism underlying MI is complex and involves inflammatory response, calcium overload, mitochondrial disorders, the production of reactive oxygen, and cell apoptosis [8, 9, 10]. Developmental endothelial locus‐1 (DEL‐1) is a secreted multifunctional domain protein secreted by endothelial and other cells and has been reported to have an anti‐inflammatory effect [11, 12, 13]. DEL‐1 may be a promising biomarker and therapeutic target for cardiovascular diseases [14]. For example, Failer et al. [15] have reported that DEL‐1 could protect from hypertension‐induced cardiovascular remodeling through immunomodulation. Moreover, Zhao et al. [16] have demonstrated that DEL‐1 deficiency could aggravate pressure overload‐induced heart failure through promoting neutrophil infiltration and neutrophil extracellular trap formation. However, little is known about whether DEL‐1 influences MI development.

In the current study, we investigated for the first time the effect of DEL‐1 on MI and elucidated its underlying mechanisms. Our findings revealed that DEL‐1 could alleviate myocardial fibrosis, inflammation, and cardiomyocyte apoptosis induced by MI via regulating the sirtuin 1 (Sirt1)/nuclear factor‐kappaB (NF‐κB) signaling pathway. Our findings on the cardioprotective effects of DEL‐1 highlight its potential as a therapeutic target for MI, particularly in populations with limited access to advanced medical interventions. DEL‐1‐based therapies may offer an affordable and accessible treatment option for patients in both developed and developing countries.

## 2 Materials and Methods

### 2.1 Animals and Experimental Protocols

Male SD rats (8 weeks, 250–280 g each) were provided by Pengyue (Jinan, China). The rats were anesthetized with intraperitoneal injection of 60 mg/kg pentobarbital sodium, and then fixed on a hardwood board in a supine position. Next, the distal 1/3 of the left anterior descending coronary artery was ligated by a 6‐0 suture. Furthermore, the rats were subjected to reperfusion for 3 h following coronary artery occlusion for half an hour. Sham group rats received the same operation but without ligation. This study was authorized by the Ethics Committee of Shandong Provincial Hospital Affiliated to Shandong First Medical University (NO. 2022‐640).

### 2.2 AAV9 Mediated DEL‐1 Delivery In Vivo

Rats were randomly divided into five groups (*n* = 5 each): Control, Sham, MI, MI + AAV‐null, and MI + AAV‐DEL‐1. One week before the MI surgery, 24 μL of adeno‐associated viral (AAV) containing either an empty vector or DEL‐1 cDNA (Genechem, Shanghai, China, 2 × 10^12^ viral genomes/mL) was injected into three points in the left ventricles.

### 2.3 Cardiac Function Detection

Two weeks after MI, rats were subjected to anesthesia and then underwent transthoracic echocardiography using a Vevo2100 high‐frequency ultrasound system (Visual Sonics Inc., Toronto, Canada). The percentage of ejection fraction (EF) or fractional shortening (FS) were measured for all rats.

### 2.4 Hemodynamics Detection

Two weeks after MI, rats were subjected to anesthesia, and the systolic blood pressure (SBP), diastolic blood pressure (DBP), and mean arterial pressure (MAP) were recorded using a physiograph connected to a catheter inserted in the right femoral artery.

### 2.5 Detection of Lactate Dehydrogenase (LDH) and Creatine Kinase‐MB (CK‐MB)

Two weeks after MI, the rats were euthanized using intraperitoneal injection of 250 mg/kg pentobarbital sodium. The heart tissues were collected, and myocardial tissue homogenates were prepared. Cardiac LDH and CK‐MB levels were detected based on the corresponding commercial kits (both supplied by Jiancheng Bioengineering Institute, Nanjing, China).

### 2.6 Histological Analysis and TUNEL Staining

Heart specimens were fixed in 10% buffered formalin, then dehydrated in ascending grades of alcohol (70%, 95%, and 99% 2 min each), and cleared with xylene. After paraffin embedding, 5‐μm‐thick sections were cut. Subsequently, we utilized H&E and Masson's trichrome for dying sections for observing the change of myocardial morphology and evaluating the collagen deposition, respectively. Images were taken with a light microscope (NIKON, Japan). The severity of the myocardial histological score was assessed as follows: normal (0), mild (1), moderate (2), severe (3), and extremely severe injury (4). The cardiomyocyte apoptosis in heart tissue was observed by using a TUNEL Apoptosis Kit (Solarbio, Beijing, China). Next, the TUNEL‐positive cells were observed by a light microscope.

### 2.7 qRT‐PCR

Total RNA was extracted from heart tissues and primary cardiomyocytes utilizing TriQuick Reagent (Solarbio, Beijing, China), and cDNA was then synthesized with HiScript IV RT SuperMix for qPCR (+gDNA wiper) (Vazyme, Nanjing, China). qRT‐PCR was performed using Taq Pro Universal SYBR qPCR Master Mix (Vazyme, Nanjing, China) on the 7900HT Fast Real‐Time PCR System (Applied Biosystems, USA). The primer sequences for the amplification of DEL‐1, TNF‐α, IL‐1β, IL‐6, and β‐actin were as follows: DEL‐1 F: 5′‐CCAGTTCGTCAAAGGGGACA‐3′, and R: 5′‐CATCCGCCAATCCTGACAGA‐3′; TNF‐α F: 5′‐GATCGGTCCCAACAAGGAGG‐3′, and R: 5′‐CTTGGTGGTTTGCTACGACG‐3′; IL‐1β F: 5′‐CAGCTTTCGACAGTGAGGAGA‐3′, and R: 5′‐TGTCGAGATGCTGCTGTGAG‐3′; IL‐6 F: 5′‐GTTTCTCTCCGCAAGAGACTTC‐3′, and R: 5′‐TGTGGGTGGTATCCTCTGTGA‐3′; β‐actin F: 5′‐ATATCGCTGCGCTCGTCGT‐3′, and R: 5′‐ CATACCCACCATCACACCCTGG‐3′.

### 2.8 Western Blot Analysis

Total proteins from heart tissues, primary cardiomyocytes, and cardiac fibroblasts were extracted using RIPA buffer (Solarbio, Beijing, China). Proteins were subjected to 10% SDS‐PAGE and transferred onto a PVDF membrane (Solarbio, Beijing, China). Following blocked, the PVDF membrane was incubated with the indicated primary antibodies (DEL‐1, 1:500, #ab190692, Abcam, UK; Collagen I, 1:1000, #ab270993, Abcam, UK; Collagen III, 1:1000, #ab184993, Abcam, UK; Bax, 1:1000, #ab32503, Abcam, UK; cleaved caspase‐3, 1:500, #9661, Cell Signaling, USA; Bcl‐2, 1:1000, #ab194583, Abcam, UK; α‐SMA, 1:1000, #55135‐1‐AP, Proteintech, Wuhan, China; p27kip1, 1:1000, #ab32034, Abcam, UK; PCNA, 1:1000, #ab92552, Abcam, UK; Sirt1, 1:1000, #ab189494, Abcam, UK; NF‐κB, 1:1000, #8242, Cell Signaling, USA; p‐NF‐κB, 1:500, #3033, Cell Signaling, USA; GAPDH, 1:5000, #10494‐1‐AP, Proteintech, Wuhan, China) at 4°C overnight. On the second day, the secondary antibody was utilized for incubating the membranes for 60 min. Protein bands were visualized utilizing an ECL kit (Solarbio, Beijing, China). The expression of proteins was normalized to GAPDH.

### 2.9 Isolation and Culture of Primary Cardiac Cardiomyocytes and Fibroblasts

The ventricle of the SD rat (1‐day old) was excised, put into pre‐cooled disassociation buffer, and cut into pieces 1 mm^3^ on ice. The small pieces were digested by 0.1% type‐II collagenase for 40 min at room temperature. The digested cell suspension was filtered through the cell filter into a 50 mL Falcon tube containing re‐suspension buffer, thoroughly mixed, and centrifuged at 200 g for 5 min. Next, the cell precipitation was re‐suspended in 5 mL of overnight medium (DMEM, M199 mixed at 4:1) and then placed in the incubator for later use. The primary myocardium and fibroblasts were isolated by Percoll: high‐density (1.017 g/mL) and low‐density (0.407 g/mL). The low‐density Percoll was slowly spread on the high‐density Percoll. The cell resuspension was slowly added to Percoll. After centrifuging at 1700 g for 30 min, two layers of suspension with clear boundaries were obtained. Subsequently, the upper and lower cells were collected and labeled as fibroblasts and cardiomyocytes in turn. Fibroblasts and cardiomyocytes were cultured in DMEM medium (Gibco, USA) supplemented with 10% fetal bovine serum and 1% streptomycin/penicillin in a humidified incubator (5% carbon dioxide, 95% atmosphere, 37°C).

### 2.10 Co‐Culture of Cardiac Cardiomyocytes and Fibroblasts

The method of co‐culture cardiomyocytes was to seed fibroblasts into inserts (8 μm pore size), and then the cardiomyocytes, transfected with pcDNA‐DEL‐1 or si‐DEL‐1, were cultured in the bottom chamber of a 24‐well culture plate and co‐cultured together.

### 2.11 Cell Treatments

Primary cardiomyocytes were cultured in DMEM medium (Gibco, USA) supplemented with 10% fetal bovine serum and 1% streptomycin/penicillin in a humidified incubator (5% CO_2_, 95% atmosphere, 37°C) for 48 h. To mimic the ischemic damage, primary cardiomyocytes were cultured in the presence of glucose‐free DMEM in a sealed hypoxic incubator with 5% CO_2_ and 95% N_2_ at 37°C for 3 h. Subsequently, cardiomyocytes were cultured in normal DMEM for 6 h in a humidified incubator (5% CO_2_, 95% atmosphere, 37°C) to simulate reoxygenation damage. To overexpress DEL‐1 or deplete Sirt1, we transfected pcDNA‐DEL‐1 or Sirt1 siRNA (si‐Sirt1) into cardiomyocytes for 48 h using Lipofectamine 3000 (Invitrogen, USA) before hypoxia treatment.

### 2.12 Cell Counting Kit‐8 (CCK‐8) Assay

Primary cardiomyocyte viability was evaluated using the CCK‐8 assay (Solarbio, Beijing, China). Shortly, 5 × 10^3^ cells were seeded into 96‐well plates. After that, 10 μL of CCK‐8 was put into every well and incubated at 37°C for 120 min. In the end, the absorbance at 450 nm was examined and recorded with a microplate reader (BIO‐RAD, USA).

### 2.13 Apoptosis Assay

Primary cardiomyocytes were prepared for assessment by suspension in an incubation buffer at a density of 1 × 10^6^ cells/mL. The cardiomyocytes were stained for 20 min in the dark utilizing Annexin V and PI (Beyotime, Shanghai, China). Finally, cell apoptosis was tested using flow cytometry with a BD FACSCalibur instrument (BD Biosciences, USA).

### 2.14 Migration Assay

Cardiac fibroblasts (2.0 × 10^5^/mL) were suspended in serum‐free medium. Cardiomyocytes transfected with pcDNA‐DEL‐1 or si‐DEL‐1 were subsequently incubated in the bottom chamber of 24‐well plates. An insert (8 μm pore size) was placed in wells for placing cardiac fibroblasts. After 24 h of co‐culture of cardiomyocytes and fibroblasts, inserts were removed from the new 24‐well culture plates containing PBS to remove unattached cells. After that, the fibroblasts were fixed in 4% paraformaldehyde for 20 min at room temperature, dyed using 0.1% crystal violet for 10 min at room temperature, and then photographed and counted under a light microscope (NIKON, Japan).

### 2.15 ELISA

The levels of IL‐1β, IL‐6, and TNF‐α in cardiomyocytes were analyzed utilizing their corresponding ELISA kits based on the instructions (Solarbio, Beijing, China).

### 2.16 Immunofluorescence Staining

The stably transfected cardiomyocytes seeded on coverslips were fixed using 4% paraformaldehyde in PBS for 20 min. The cell sections or paraffin‐embedded heart sections were incubated with primary antibodies (DEL‐1, 1:50, #ab190692, Abcam, UK; Sirt1, #ab189494, 1:100, Abcam, UK; p‐NF‐κB, #3033, 1:100, Cell Signaling, USA) overnight at 4°C, followed by the fluorescent secondary antibody at a dilution of 1:1000 incubation for 1 h at room temperature. Subsequently, coverslips were mounted using an anti‐fade mounting solution supplemented with DAPI. Finally, the sections were observed and photographed utilizing a fluorescence microscope (NIKON, Japan).

### 2.17 Statistical Methods

All data are shown in terms of the mean ± SD. GraphPad Prism 8.0 was used for statistical analysis. Differences were analyzed using Student's *t*‐test or one‐way ANOVA with Tukey's post hoc test. *p*‐values of less than 0.05 were considered significant.

## 3 Results

### 3.1 DEL‐1 Expression Was Downregulated in MI Rats

**Figure 1:**
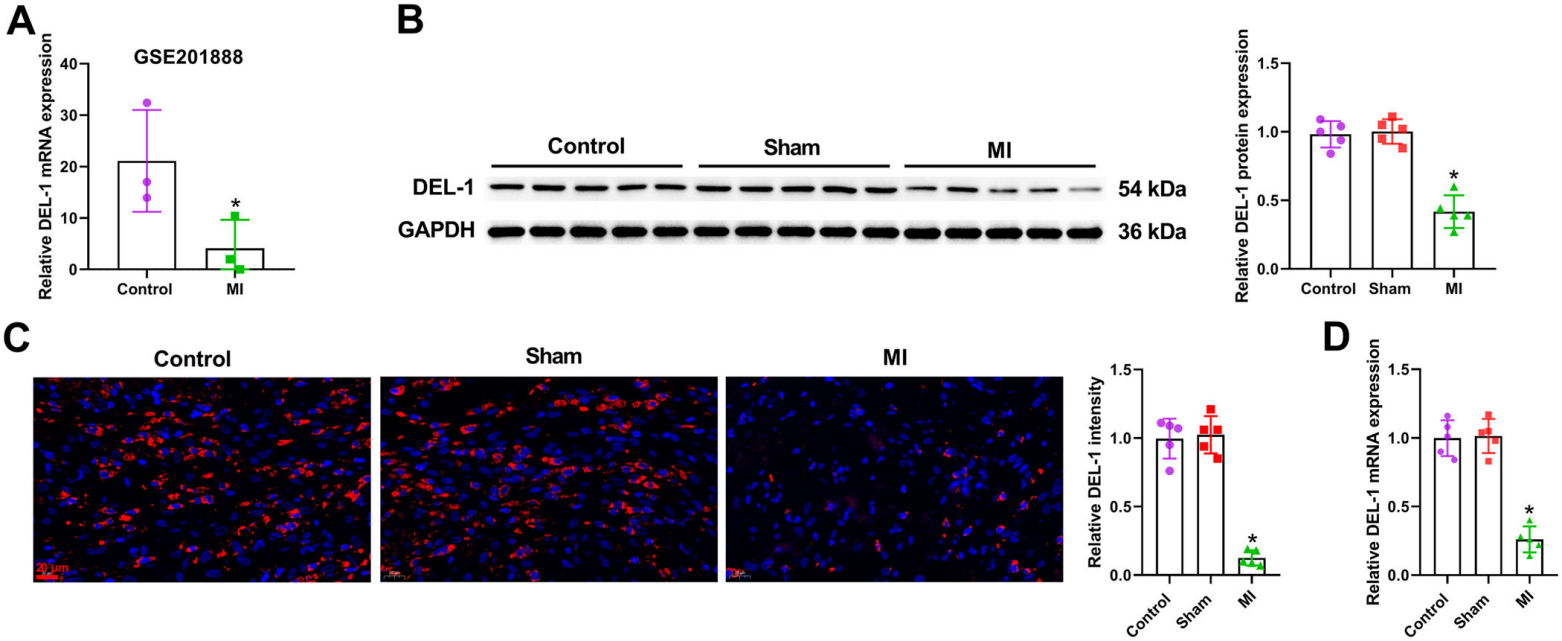
DEL‐1 expression was downregulated in MI rats. (A) GSE201888 showed that the DEL‐1 mRNA level was downregulated in MI (*n* = 3). (B) Western blot analysis, (C) immunofluorescence, and (D) qRT‐PCR were utilized for detecting DEL‐1 expression in MI rats (*n* = 5). Scale bar: 20 μm (×400). Data were displayed as mean ± SD. Statistical analyses were performed using Student's *t*‐test or one‐way ANOVA with Tukey's post hoc test. *p* < 0.05 was considered to indicate a statistically significant difference. (A) *p < 0.05 versus Control group. (B–D) *p < 0.05 versus Sham group.

The data of GSE201888 showed that DEL‐1mRNA level was decreased in MI (Figure 1A). At the same time, the results of Western blot analysis, immunofluorescence and qRT‐PCR demonstrated that the expression of DEL‐1 was significantly downregulated after MI model establishment (Figure 1B–D).

### 3.2 DEL‐1 Overexpression Alleviated Heart Damage in MI Rats

**Figure 2:**
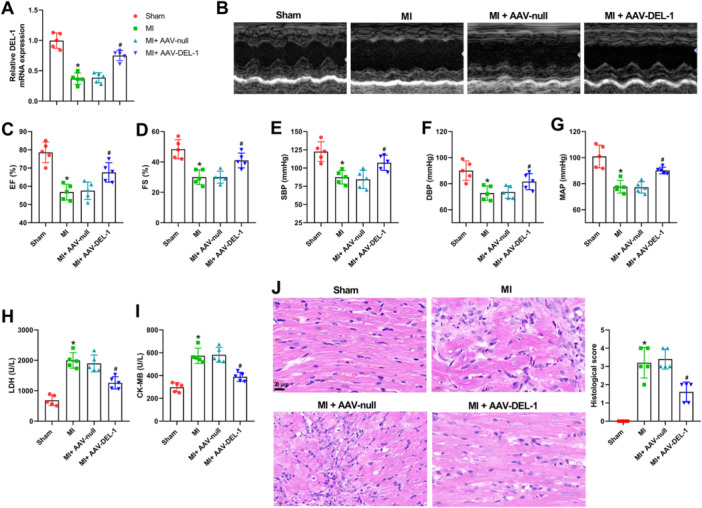
DEL‐1 overexpression alleviated heart damage in MI rats. (A) qRT‐PCR was used to detect DEL‐1 mRNA levels. (B) Representative echocardiographic images. (C) EF and (D) FS were quantified. The measurement of SBP (E), DBP (F), and MAP (G). (H) Expression level of LDH in heart tissues. (I) Expression level of CK‐MB in heart tissues. (J) HE staining was performed to examine pathological and morphological changes in myocardial tissue. Scale bar: 20 μm (×400). *n* = 5. Data were displayed as mean ± SD. Statistical analyses were performed using one‐way ANOVA with Tukey's post hoc test. *p* < 0.05 was considered to indicate a statistically significant difference. *p < 0.05 versus Sham group, ^#^ *p* < 0.05 versus MI + AAV‐null group.

To determine whether DEL‐1 has a cardioprotective function in vivo, DEL‐1 gain‐of‐function studies were performed. We found an obvious overexpression of DEL‐1 in heart tissues after intramyocardial injection of AAV‐DEL‐1 in MI rats (Figure 2A). Echocardiography results demonstrated that the indicators of left ventricular systolic function (EF and FS) were significantly reduced after MI (Figure 2B–D). DEL‐1 overexpression remarkedly increased the above parameters in MI rats (Figure 2B–D). Moreover, compared with the Sham group, SBP, DBP, and MAP were significantly decreased in the MI group (Figure 2E–G). There were recoveries in SBP, DBP, and MAP in the MI + AAV‐DEL‐1 group compared to the MI + AAV‐null group (Figure 2E–G). As Figure 2H,I showed, the levels of LDH and CK‐MB were elevated in the MI group, but reduced after the treatment of DEL‐1 overexpression. Moreover, HE staining indicated that overexpression of DEL‐1 significantly relieved the myocardial injury induced by MI (Figure 2J).

### 3.3 DEL‐1 Overexpression Alleviated Myocardial Fibrosis, Inflammation, and Cardiomyocyte Apoptosis in MI Rats

**Figure 3:**
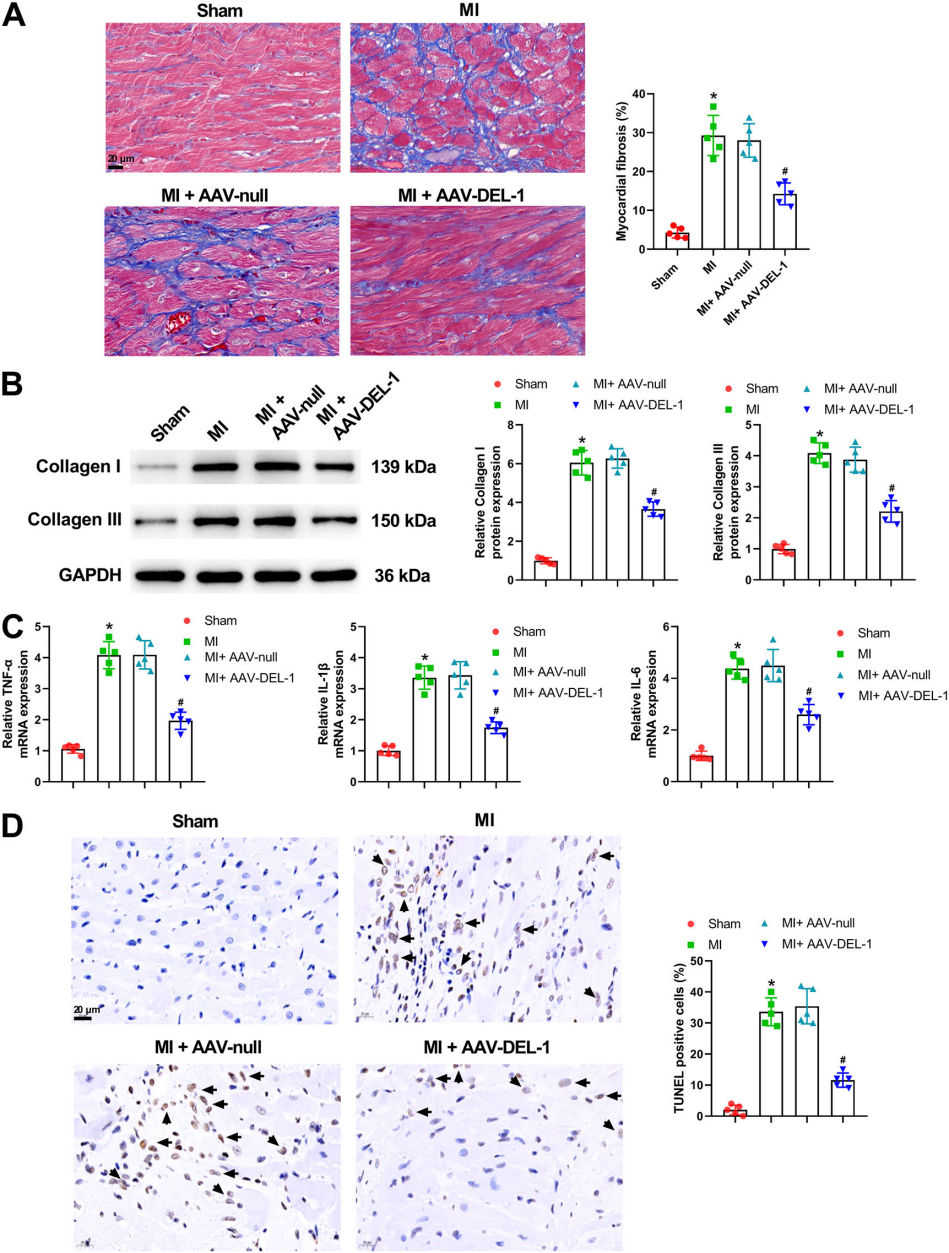
DEL‐1 overexpression alleviated myocardial fibrosis, inflammation, and cardiomyocyte apoptosis in MI rats. (A) Masson's trichrome staining of myocardial sections. (B) Western blot analysis was applied for detecting Collagen I and Collagen III expression. (C) qRT‐PCR was applied for assessing TNF‐α, IL‐1β, and IL‐6 mRNA expression. (D) Representative images of myocardial sections stained with TUNEL. Scale bar: 20 μm (×400). *n* = 5. Data were displayed as mean ± SD. Statistical analyses were performed using one‐way ANOVA with Tukey's post hoc test. *p* < 0.05 was considered to indicate a statistically significant difference. *p < 0.05 versus Sham group, ^#^ *p* < 0.05 versus MI + AAV‐null group.

Masson's trichrome staining demonstrated that the greater fibrosis was observed in the MI group compared with the sham group (Figure 3A). Meanwhile, DEL‐1 overexpression significantly attenuated the increased myocardial fibrosis induced by MI (Figure 3A). There were higher levels of collagen I and collagen III in the heart tissues of MI rats compared to rats in the sham group (Figure 3B). Moreover, the results of qRT‐PCR showed that when compared with the Sham group, the mRNA level of TNF‐α, IL‐1β, and IL‐6 was notably upregulated in the MI group rats (Figure 3C). The upregulation of DEL‐1 markedly reversed the increased above indicators in MI rats (Figure 3B,C). Additionally, the cardiomyocyte apoptosis significantly increased after MI, and the increased cardiomyocyte apoptosis was markedly reduced by the overexpression of DEL‐1 (Figure 3D). All these results implied that DEL‐1 overexpression could alleviate myocardial fibrosis, inflammation, and cardiomyocyte apoptosis in MI rats.

### 3.4 DEL‐1 Overexpression Alleviated Cardiomyocyte Apoptosis and Inflammation

**Figure 4:**
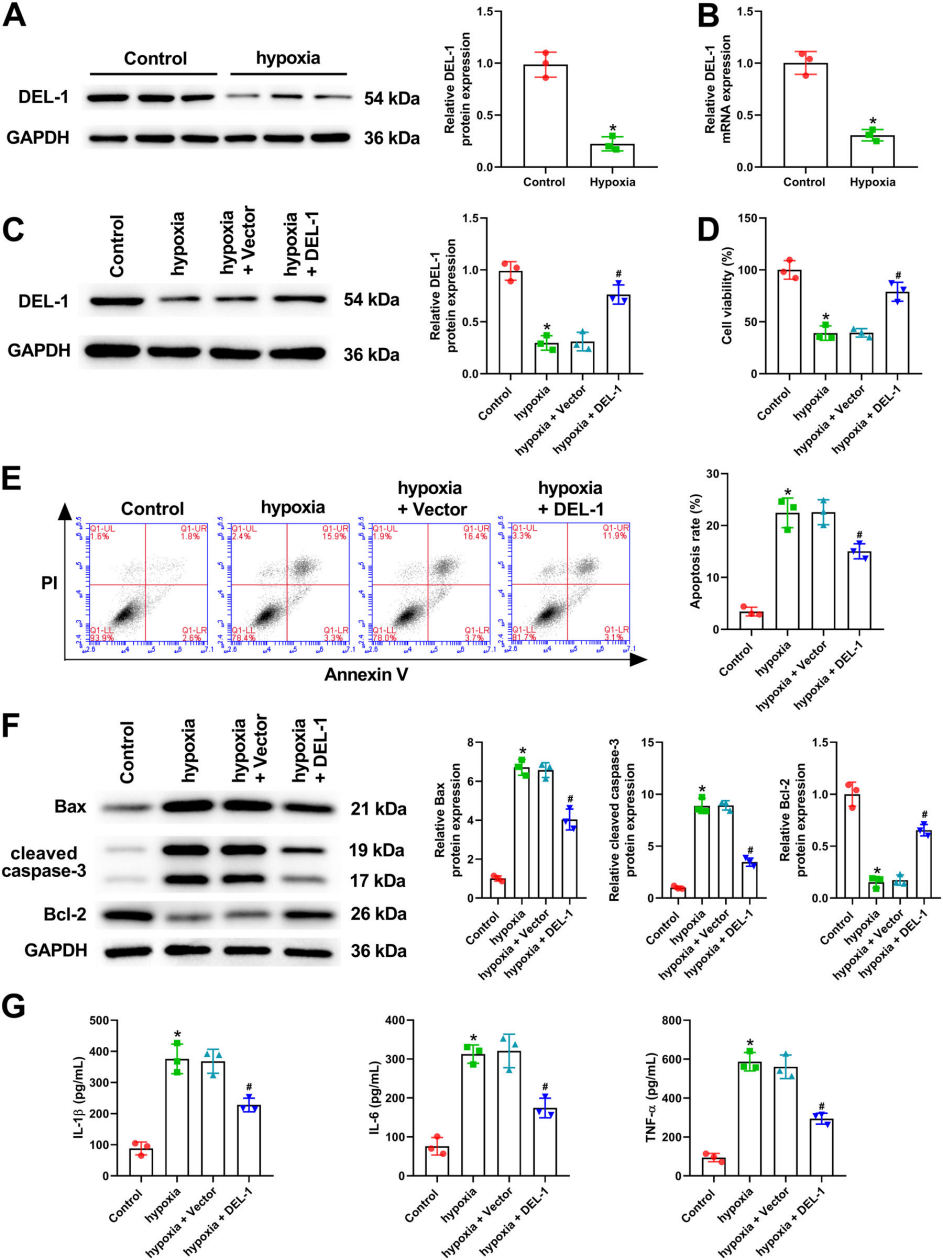
DEL‐1 overexpression alleviated cardiomyocyte apoptosis and inflammation. (A) Western blot analysis and (B) qRT‐PCR for measuring DEL‐1 level in cardiomyocytes. After transfected with DEL‐1 overexpression vector, (C) Western blot analysis was employed for measuring DEL‐1 level in cardiomyocytes; (D) CCK‐8 assay was applied for evaluating cell viability; (E) flow cytometry was utilized for evaluating cell apoptosis; (F) Western blot analysis was employed for assessing the expression of apoptosis‐related proteins; (G) ELISA was applied for assessing IL‐1β, IL‐6, and TNF‐α level. *n* = 3. Data were displayed as mean ± SD. Statistical analyses were performed using Student's *t*‐test or one‐way ANOVA with Tukey's post hoc test. *p* < 0.05 was considered to indicate a statistically significant difference. *p < 0.05 versus Control group, ^#^ *p* < 0.05 versus hypoxia + Vector group.

As seen in Figure 4A,B, DEL‐1 was downregulated in hypoxia‐treated cardiomyocytes. To investigate the effect of DEL‐1 on MI in vitro, we transfected pcDNA‐DEL‐1 into cardiomyocytes, and then detected the transfection efficiency using Western blot analysis (Figure 4C). Cardiomyocytes' viability was suppressed following hypoxia (Figure 4D). On the contrary, DEL‐1 overexpression significantly reversed the decreased cell viability induced by hypoxia (Figure 4D). Besides, the data of flow cytometry demonstrated cardiomyocytes apoptosis was remarkably elevated following hypoxia, and the increased apoptosis was attenuated by DEL‐1 overexpression (Figure 4E). In addition, we also explored the expression of apoptosis‐related proteins using Western blot analysis, and observed that the upregulated expression of Bax and cleaved caspase‐3, and the downregulated expression of Bcl‐2 induced by hypoxia in cardiomyocytes were markedly reversed by DEL‐1 overexpression (Figure 4F). Moreover, we also confirmed that the elevated level of IL‐1β, IL‐6, and TNF‐α caused by hypoxia was significantly reversed by DEL‐1 overexpression (Figure 4G). In summary, DEL‐1 overexpression could alleviate cardiomyocyte apoptosis and inflammation in vitro.

### 3.5 DEL‐1 Secreted by Cardiomyocytes Activated Cardiac Fibroblasts Through Paracrine Signaling

**Figure 5:**
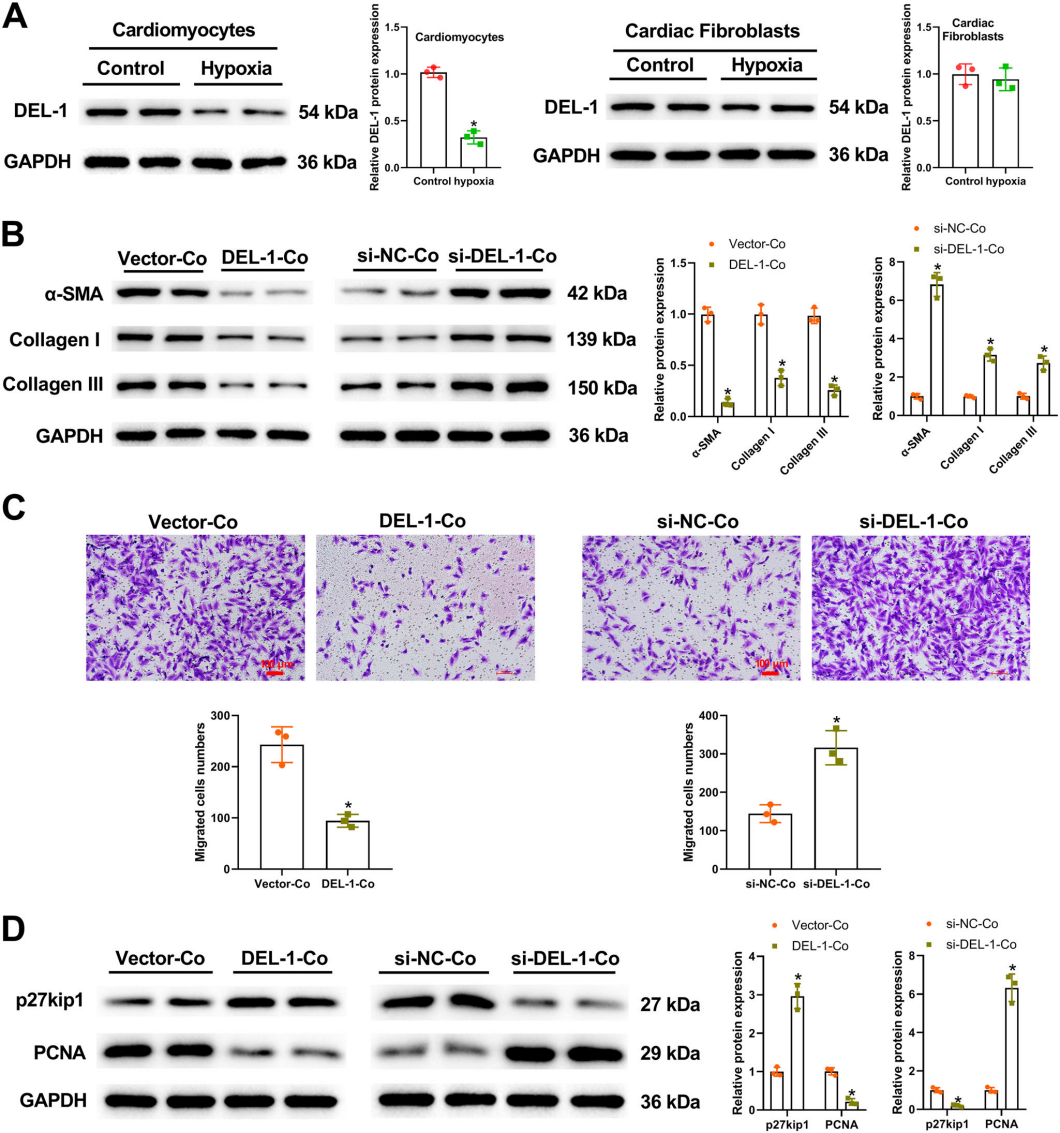
DEL‐1 secreted by cardiomyocytes activated cardiac fibroblasts through paracrine signaling. (A) After hypoxia treatment, the expression of DEL‐1 was detected using Western blot analysis in cardiomyocytes and cardiac fibroblasts. (B) α‐SMA, Collagen I, and Collagen III expressions in cardiac fibroblasts co‐cultured with cardiomyocytes transfected with pcDNA‐DEL‐1 (DEL‐1‐Co) or si‐DEL‐1 (si‐DEL‐1‐Co) were measured using Western blot analysis. (C) The migrative ability of cardiac fibroblasts was assessed by transwell assay. (D) p27kip1 and PCNA expressions were evaluated utilizing Western blot analysis. Scale bar: 100 μm (×100). *n* = 3. Data were displayed as mean ± SD. Statistical analyses were performed using Student's *t*‐test. *p* < 0.05 was considered to indicate a statistically significant difference. *p* < 0.05 was considered to indicate a statistically significant difference. (A) *p < 0.05 versus Control group. (B–D) *p < 0.05 versus Vector‐Co or si‐NC‐Co group.

As seen in Figure 5A, the expression change of DEL‐1 in cardiomyocytes was more obvious compared with that in cardiac fibroblasts under hypoxia. Figure 5B demonstrated that α‐SMA, Collagen I, and Collagen III expression were significantly decreased in cardiac fibroblasts co‐cultured with cardiomyocytes transfected with pcDNA‐DEL‐1, but increased in cardiac fibroblasts co‐cultured with si‐DEL‐1‐Co. Transwell assay demonstrated that the migration of cardiac fibroblasts was inhibited when cardiac fibroblasts were co‐cultured with cardiomyocytes transfected with pcDNA‐DEL‐1 (Figure 5C). On the contrary, their migration was promoted when they co‐cultured with cardiomyocytes transfected with si‐DEL‐1 (Figure 5C). The results of Western blot analysis showed that the increased expression of p27kip1 and decreased expression of PCNA were observed in cardiac fibroblasts co‐cultured with cardiomyocytes transfected with pcDNA‐DEL‐1 (Figure 5D). On the contrary, p27kip1 expression was reduced, but PCNA expression was elevated in cardiac fibroblasts co‐cultured with si‐DEL‐1‐Co (Figure 5D). Overall, DEL‐1 secreted by cardiomyocytes could activate cardiac fibroblasts through paracrine signaling.

### 3.6 DEL‐1 Affected the Sirt1/NF‐κB Pathway In Vitro and In Vivo

**Figure 6:**
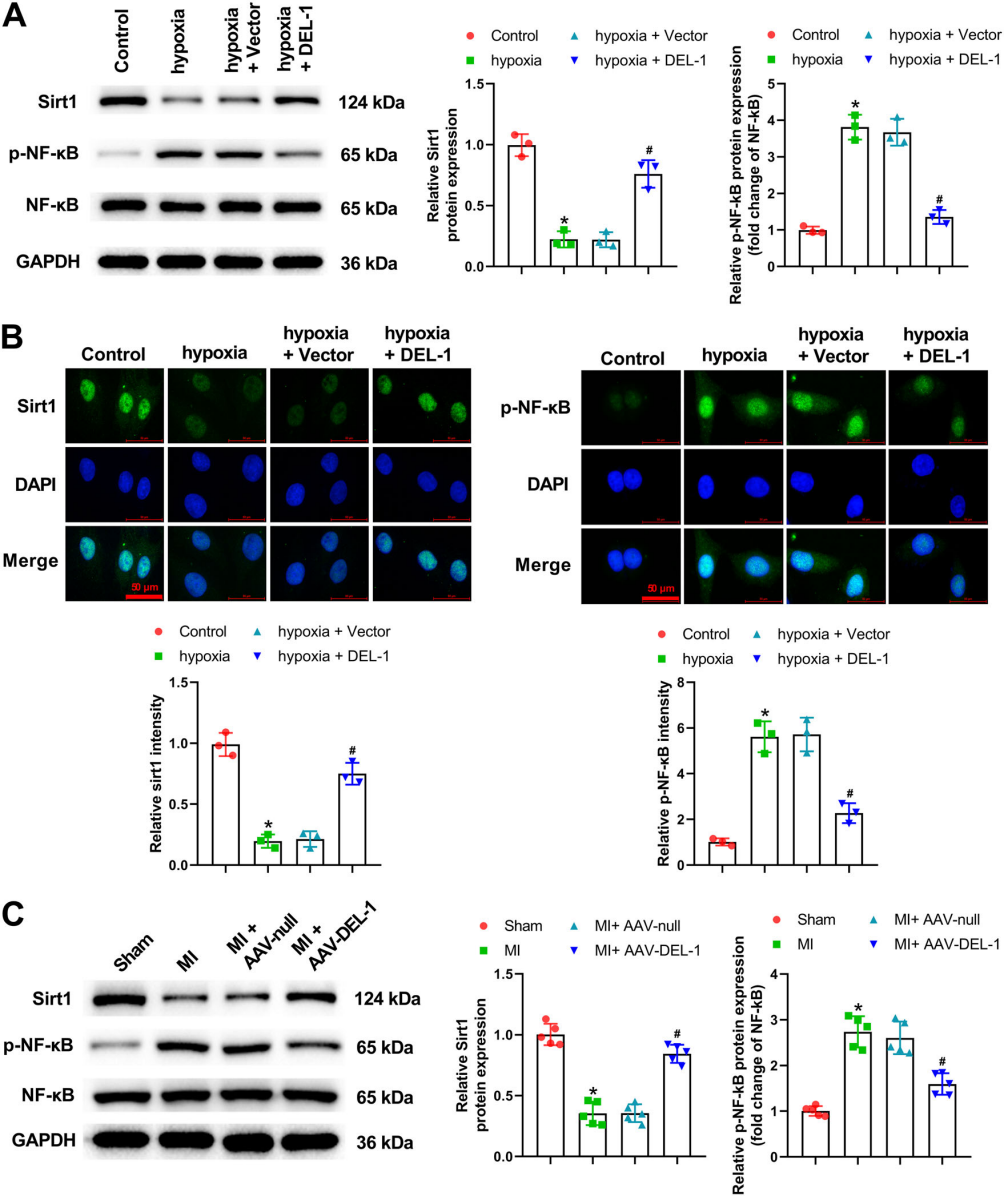
DEL‐1 regulated the Sirt1/NF‐κB pathway in vitro and in vivo. (A) Sirt1, p‐NF‐κB, and NF‐κB expression in cardiomyocytes was measured utilizing Western blot analysis. (B) Sirt1 and p‐NF‐κB expression in cardiomyocytes was measured by immunofluorescence staining. (C) Sirt1, p‐NF‐κB, and NF‐κB expression in heart tissues was detected utilizing Western blot analysis. Scale bar: 50 μm (×400). *n* = 3. Data were displayed as mean ± SD. Statistical analyses were performed using one‐way ANOVA with Tukey's post hoc test. *p* < 0.05 was considered to indicate a statistically significant difference. (A and B) *p < 0.05 versus Control group, ^#^ *p* < 0.05 versus hypoxia + Vector group. (C) *p < 0.05 versus Sham group, ^#^ *p* < 0.05 versus MI + AAV‐null group.

As shown in Figure 6A,B, Sirt1 level in cardiomyocytes was decreased after hypoxia, but the expression of p‐NF‐κB was notably increased. In addition, DEL‐1 overexpression markedly reversed the abnormal expression of Sirt1 and p‐NF‐κB caused by hypoxia in cardiomyocytes (Figure 6A,B). In MI rats, the downregulated Sirt1 expression and upregulated p‐NF‐κB expression were observed (Figure 6C). Similarly, DEL‐1 overexpression notably attenuated the abnormal expression of Sirt1 and p‐NF‐κB caused by MI in rats (Figure 6C).

### 3.7 The Downregulation of Sirt1 Reversed the Effect of DEL‐1 on Cardiomyocyte Apoptosis and Inflammation

**Figure 7:**
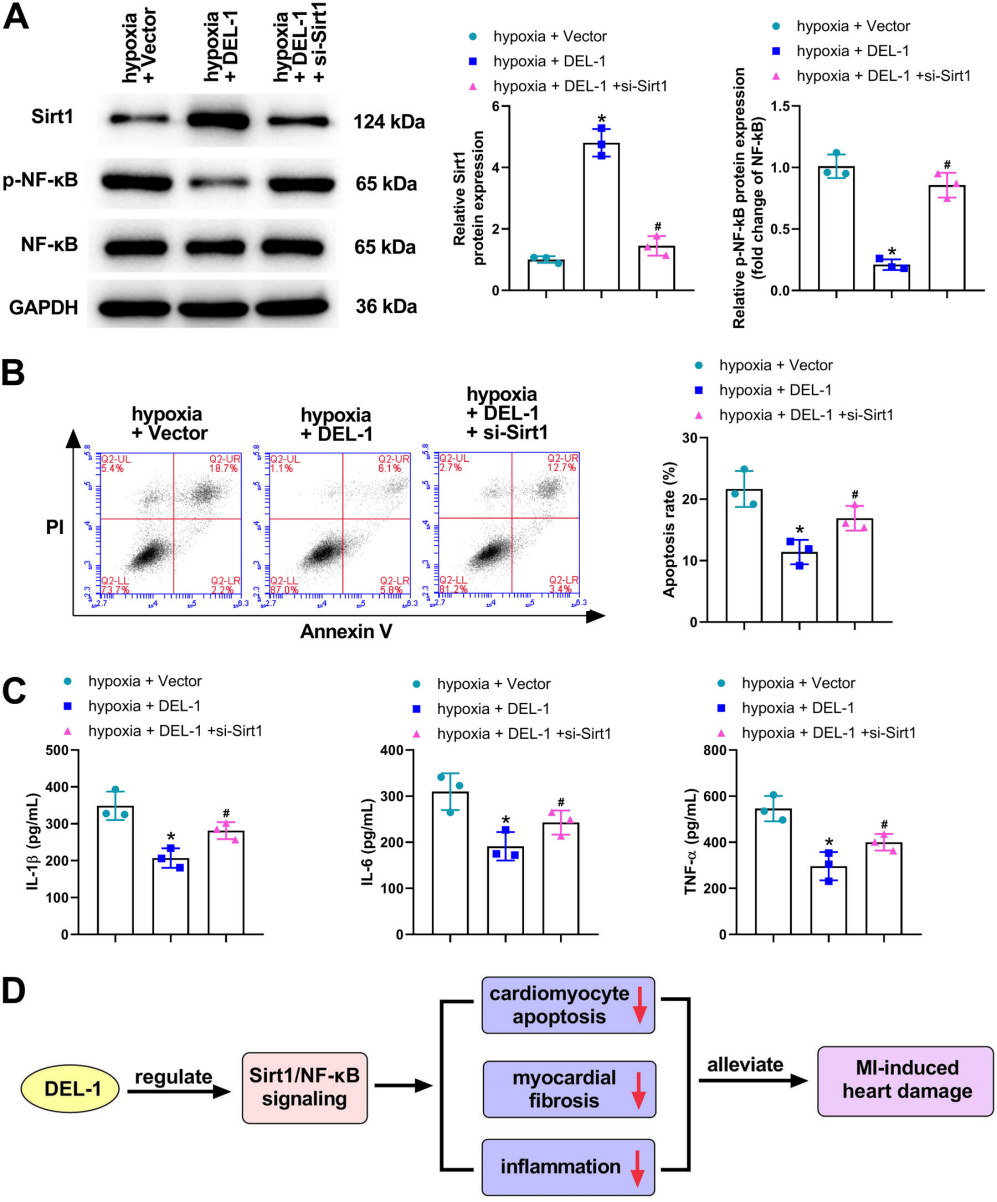
The downregulation of Sirt1 reversed the effect of DEL‐1 on cardiomyocyte apoptosis and inflammation. (A) Sirt1, p‐NF‐κB, and NF‐κB expression in cardiomyocytes was measured utilizing Western blot analysis. (B) Flow cytometry was utilized for evaluating cell apoptosis. (C) ELISA was applied for assessing IL‐1β, IL‐6, and TNF‐α levels in cardiomyocytes. (D) The mechanism diagram of this study. *n* = 3. Data were displayed as mean ± SD. Statistical analyses were performed using one‐way ANOVA with Tukey's post hoc test. *p* < 0.05 was considered to indicate a statistically significant difference. *p < 0.05 versus hypoxia + Vector group, ^#^ *p* < 0.05 versus hypoxia + DEL‐1 group.

As seen in Figure 7A, the expression of Sirt1 was elevated in hypoxia + DEL‐1 group relative to hypoxia + Vector group, but the expression of p‐NF‐κB was markedly downregulated. When compared with hypoxia + DEL‐1 group, the expression of Sirt1 was decreased in hypoxia + DEL‐1 + si‐Sirt1 group, while the expression of p‐NF‐κB was significantly increased (Figure 7A). Besides, DEL‐1 overexpression significantly inhibited the apoptosis induced by hypoxia (Figure 7B). Nevertheless, the silence of Sirt1 remarkably reversed the decreased apoptosis caused by DEL‐1 overexpression (Figure 7B). Moreover, we also found that the silence of Sirt1 significantly attenuated the downregulated level of IL‐1β, IL‐6, and TNF‐α caused by DEL‐1 overexpression in cardiomyocytes (Figure 7C). In summary, the silence of Sirt1 could reverse the inhibitory effect of DEL‐1 overexpression on cardiomyocyte apoptosis and inflammation.

## 4 Discussion

MI is a cardiovascular disease which is harmful to human health and imposes a heavy burden on society. Although there is continuous improvement in the treatment of MI, the mortality and morbidity rates of MI remain high [17]. In this study, we demonstrated that DEL‐1 could alleviate myocardial fibrosis, inflammation, and cardiomyocyte apoptosis in MI in vitro and in vivo via regulating the Sirt1/NF‐κB signaling pathway.

DEL‐1 is considered to be a homeostatic factor controlling harmful inflammatory responses [18, 19]. At present, DEL‐1 has been reported to participate in multiple diseases. For instance, the overexpression of DEL‐1 can inhibit porphyromonas gingivalis‐induced gingival inflammation in vivo and in vitro [20]. Yuh et al. [21] have suggested that DEL‐1 could promote osteogenic differentiation and bone regeneration via activating the β3 integrin‐FAK‐ERK1/2‐RUNX2 pathway. Wang et al. [22] report that stromal cell‐derived DEL‐1 could inhibit Tfh cell activation and inflammatory arthritis. Shin et al. [23] demonstrate that DEL‐1 could restrain osteoclastogenesis and repress inflammatory bone loss in nonhuman primates. Interestingly, increasing evidence has proved that DEL‐1 can play important roles in cardiovascular diseases via modulating immune system homeostasis [14, 16]. Hence, we inferred that DEL‐1 also has a role in regulating MI. First, we investigated the expression of DEL‐1 in MI and found that DEL‐1 expression was downregulated in MI. Cardiomyocyte apoptosis can exert important roles at multiple points in the evolution of MI [24]. In addition, apoptosis is also reported to participate in remodeling and development of heart failure after MI [24]. After MI, myocardial apoptosis causes inflammatory responses [25]. Cytokines are released from the infarcted myocardium, and the secreted cytokines can further stimulate infiltrating leukocytes and endothelial cells to release pro‐inflammatory cytokines, which further aggravate myocardial injury [26, 27]. In our study, we demonstrated that DEL‐1 overexpression could alleviate myocardial fibrosis, inflammation, and cardiomyocyte apoptosis in the MI rat model and cell model.

Sirt1 has been considered to participate in cell survival, cell death, and modulation of metabolism [28, 29, 30]. It is reported that activation of the Sirt1 pathway can significantly attenuate MI through decreasing oxidative injury, apoptosis, and inflammation responses [31, 32]. Besides, multiple evidence have supported Sirt1 function in fibrosis in several organs, such as the liver and heart [33, 34, 35]. SIRT1 can regulate NF‐κB deacetylation, which leads to anti‐inflammatory functions, further prevents NF‐κB nuclear translocation, and suppresses NF‐κB activation [36]. Importantly, NF‐κB is a transcription factor, which is closely related to the immune responses [37], and has been demonstrated to be a significant factor in the molecular mechanisms that lay on the basis of MI [38]. Tu et al. [39] have proved that the SIRT1 activator exerts anti‐inflammatory effects via NF‐κB during MI. Shan et al. [40] have indicated that Rap1GAP exacerbates MI through modulating the Sirt1/NF‐κB signaling pathway. In addition, a study by Cheng et al. [41] has revealed that DEL‐1 overexpression could promote spinal cord injury recovery in mice through regulating the Sirt1/SERCA2 pathway. Therefore, we investigated whether DEL‐1 overexpression alleviated myocardial fibrosis, inflammation, and cardiomyocyte apoptosis in MI through regulating the Sirt1/NF‐κB pathway. As expected, our experimental results confirmed the above hypothesis.

In conclusion, we found that DEL‐1 expression was downregulated in MI. In addition, we demonstrated that DEL‐1 could alleviate myocardial fibrosis, inflammation, and cardiomyocyte apoptosis in MI via regulating the Sirt1/NF‐κB signaling pathway (Figure 7D). In view of the accumulated results, overexpression of DEL‐1 appears as a promising therapeutic strategy for treating MI. Moreover, the data in this study are expected to provide a reliable theoretical basis for clinical prevention of MI and the search for effective therapeutic drugs. Of course, our study also has some limitations. First, we only conducted preliminary research on animals, and further histological studies related to humans are needed. Second, the mechanism needs to be further explored to provide stronger support for our conclusion. Third, we will explore the per se effects of DEL‐1 in healthy controls in future research.

## Author Contributions

Conception and design: Bin Hu. Administrative support: Bin Hu. Provision of study materials or patients: Hao Lin. Collection and assembly of data: Bin Hu, Wenlong Zhang, and Wan‐Cheng Yu. Data analysis and interpretation: Hao Lin. Manuscript writing: Bin Hu and Hao Lin. Final approval of manuscript: All authors.

## Ethics Statement

The animal IRB of Shandong Provincial Hospital Affiliated to Shandong First Medical University approved the experimental study (NO. 2022‐640). All studies follow ARRIVE guidelines.

## Consent

The authors have nothing to report.

## Conflicts of Interest

The authors declare no conflicts of interest.

## Data Availability

The data sets used and analyzed during the current study are available from the corresponding author on reasonable request.
